# Detection of mycoplasma in contaminated mammalian cell culture using FTIR microspectroscopy

**DOI:** 10.1007/s00216-018-0987-9

**Published:** 2018-03-17

**Authors:** Katia Wehbe, Marzia Vezzalini, Gianfelice Cinque

**Affiliations:** 1 Diamond Light Source, Harwell Science and Innovation Campus, Didcot, Oxfordshire OX11 0DE UK; 2 Department of Medicine, General Pathology Section, University of Verona, Strada Le Grazie, 8, 37134 Verona, Italy

**Keywords:** Mycoplasma, Cell infection, FTIR microspectroscopy, Focal plane array, PCA, HCA

## Abstract

Mycoplasma contamination represents a significant problem to the culture of mammalian cells used for research as it can cause disastrous effects on eukaryotic cells by altering cellular parameters leading to unreliable experimental results. Mycoplasma cells are very small bacteria therefore they cannot be detected by visual inspection using a visible light microscope and, thus, can remain unnoticed in the cell cultures for long periods. The detection techniques used nowadays to reveal mycoplasma contamination are time consuming and expensive with each having significant drawbacks. The ideal detection should be simple to perform with minimal preparation time, rapid, inexpensive, and sensitive. To our knowledge, for the first time, we employed Fourier transform infrared (FTIR) microspectroscopy to investigate whether we can differentiate between control cells and the same cells which have been infected with mycoplasmas during the culturing process. Chemometric methods such as HCA and PCA were used for the data analysis in order to detect spectral differences between control and intentionally infected cells, and spectral markers were revealed even at low contamination level. The preliminary results showed that FTIR has the potential to be used in the future as a reliable complementary detection technique for mycoplasma-infected cells.

**Graphical abstract:**
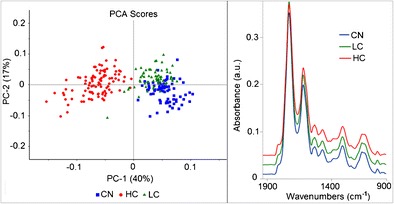
FTIR microspectroscopy is able to differentiate between mycoplasma infected cells (LC for low contamination and HC for high contamination) and control non-infected cells (CN).

FTIR microspectroscopy is able to differentiate between mycoplasma infected cells (LC for low contamination and HC for high contamination) and control non-infected cells (CN).

## Introduction

Mycoplasmas are types of bacteria of class *Mollicutes* that lack a cell wall which makes them unaffected by common antibiotics. Mycoplasma contamination is a significant problem to the culture of mammalian cells used for research and the rate could be as high as 70% [1]. The contamination can cause disastrous effects on eukaryotic cells as it tends to alter the cells at a molecular level and compromises the value of the contaminated cell lines in providing accurate data for life science research. It can induce alterations in cellular parameters (e.g., chromosome aberrations, changes in metabolism and cell growth) leading to unreliable experimental results and potentially unsafe biological products [2, 3]. In cell culture laboratories, infection usually occurs with the same mycoplasma species, and this proves that mycoplasma infections are often spread from one culture to another [4, 5]. The sources of mycoplasma contamination in the laboratory are very challenging to completely control. Since certain mycoplasma species are found on human skin, they can be introduced in the cultures through poor aseptic technique. They can also originate from contaminated supplements such as fetal bovine serum (FBS) and certainly from other contaminated cell cultures. For these reasons, good aseptic technique should be followed and new cell lines received from other laboratories should be considered suspicious and quarantined till the proof of mycoplasma absence.

Mycoplasma cells are very small (less than 1 μm); therefore, they cannot be detected by visual inspection using a visible light microscope and, thus, can remain unnoticed in the cell cultures for long periods. Most if not all of the detection techniques used as DNA probe, PCR, fluorescent DNA staining (with DAPI or Hoechst), microbiological culture or enzyme-linked immunosorbent assays (ELISA), and immunoblotting are time consuming and expensive with each having significant drawbacks. The ideal detection should be simple to perform with minimal preparation time, rapid, inexpensive, and sensitive and can be used to test different cell cultures on a regular basis. Unfortunately, mycoplasma assays, used nowadays, have some of these characteristics but not all. Even the polymerase chain reaction (PCR) (detection technique based on the amplification of the mycoplasma DNA in the cell culture supernatant followed by its visualization using gel electrophoresis), which is known to be the most reliable assay, has drawbacks: it is complex, time consuming, and suffers from false positive and negative results if performed inadequately [6].

Compared to other molecular and imaging techniques used nowadays, FTIR microspectroscopy has many advantages (i.e., better resolution of few micrometers, less expensive, less time consuming, sensitive, and reproducible). The use of FTIR microspectroscopy for studying biological samples is a wide and active area of research and became very common especially in the last two decades thanks to conventional instruments for imaging or synchrotron radiation IR for single cell analysis. IR spectral differences have been reported on many biological samples from cancerous and normal cells, cells in different growth stages or different environments, studying the effect of drugs on cells, and plant cells [7–20] to bacterial and parasite identification [19, 21–23] and so on. Parallel techniques such as SERS (surface-enhanced Raman scattering) were also used to evaluate nanomaterials cytotoxicity on living cells and the changes they induced [24, 25].

To our knowledge, there is no previous study reported in the literature that tried to compare/detect mycoplasma contamination in cultured mammalian cells. The only study found to analyze and characterize Mollicutes by IR spectroscopy done by Melin et al. [26] compared different strains of Mollicutes (including mycoplasma) to attempt a spectral characterization based on the biomolecular structures. It did not involve any cell culture or intentional infection of cells with mycoplasma species during the culture process as presented in this study.

FTIR imaging via a focal plane array detector (FPA) using a global source was employed in this study as it is advantageous for fast acquisition and good spectral quality necessary for cell analysis, hence considered as an ideal tool for quickly investigating the biochemical difference between infected and non-infected cells with mycoplasma for a first screening step. The results obtained by FTIR are compared with PCR technique and a biochemical test for correlation. The purpose of the present preliminary study was to introduce FTIR spectroscopy method for mycoplasma detection in cell culture as a first go. Further research and samples will have to be studied to test the specificity in different mycoplasma species.

## Materials and methods

### Mycoplasma preparation

A supernatant from a hybridoma culture (Hybridoma B lymphocyte, R4-6A2 (ATCC number HB-170)) contaminated by mycoplasma was collected and confirmed with the MycoAlert™ PLUS Mycoplasma Detection Kit (Lonza, Basel, Switzerland) to be positive. A test with a serial dilution of this medium was performed and verified that there was a detectable contamination even to a 1:128 dilution. The medium was sterilized with a 0.22-μM filter to make sure to use a bacteria-free medium culture (mycoplasma are smaller than common bacteria, typically between 0.1–0.8 μm in diameter, so a fair part of them should pass through the mesh). Ten percent of FBS and 2 mM GlutaMax (Gibco by Life Technologies) were added to this filtered medium and a second MycoAlert™ test was performed to check again the positivity (Table 1). Then a volume of the not diluted medium was used to infect DBTRG cells (Myc+++) and a dilution at 100 times of this medium was prepared to make a low contaminated cell population (Myc+).

**Table 1:** MycoAlert™ results on supernatant from hybridoma contaminated with mycoplasma

Dilution	Supernatant from HMC (not filtered)	Result interpretation for MC	Supernatant from HMC (filtered)	Result interpretation for MC
Not diluted	21.07	Positive	18.65	Positive
1:2	12.51	Positive	9.74	Positive
1:4	10.71	Positive	8.53	Positive
1:8	7.84	Positive	6.23	Positive
1:16	5.50	Positive	3.86	Positive
1:32	3.52	Positive	2.48	Positive
1:64	2.82	Positive	1.45	Positive
1:100	2.34	Positive	1.16	Borderline
1:128	1.95	Positive	1.04	Borderline
Negative ctrl	0.31	Negative	0.29	Negative
Ratio interpretation				
< 1 negative for mycoplasma				
1–1.2 borderline: quarantine cells and re-test in 24 h				
> 1.2 positive for mycoplasma contamination				

*HMC* hybridoma mycoplasma contaminated, *MC* mycoplasma contamination

### Cell preparation

**Fig. 1:**
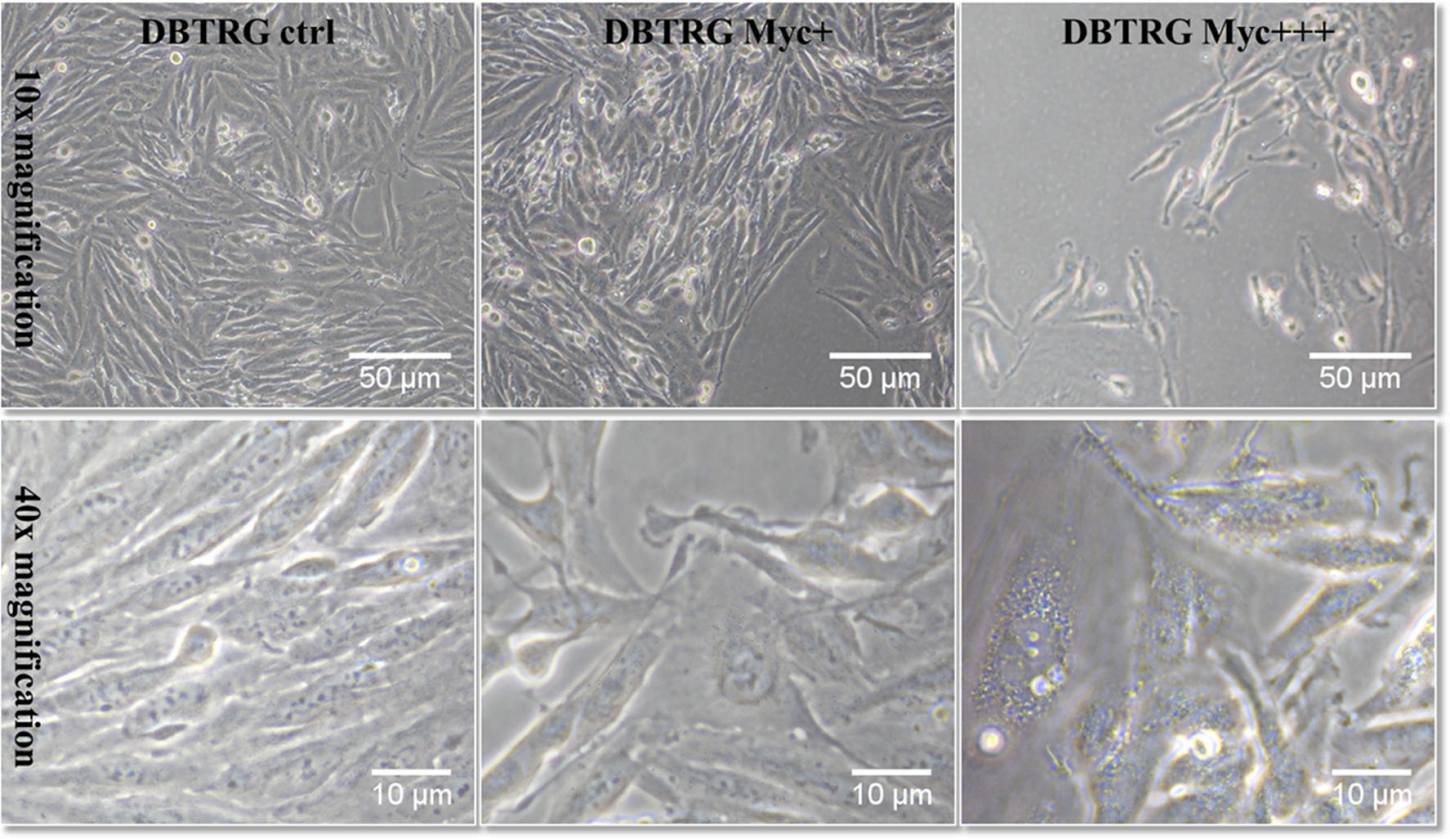
DBTRG cells; control versus contaminated cells after 24 h mycoplasma infection in T25 culture flasks (supernatant Myc+ corresponds to dilution 1:100 supernatant Myc+++) shown using an inverted microscope

DBTRG cells (a human brain glioblastoma cell line, ATCC number CRL-2020) were seeded in T25 culture flasks at a density of 1 × 10^6^ cells/ml and cultured in RPMI 1640 medium supplemented with 10% FBS, 2 mM GlutaMax, (all from Gibco, Invitrogen). Cells were maintained in a humidified atmosphere in a 37 °C incubator supplied with 5% CO_2_ to reach 70–80% confluence. They were then infected with the mycoplasma suspension (*bovis* species, identified by PCR and selective tests) during the culturing process. Three cell categories were prepared in different concentrations to have non-infected cells acting as control, low contaminated (Myc+) and high contaminated cells (Myc+++). Cells were kept to grow with or without mycoplasma for 24 h in two separate incubators to avoid contaminating the non-infected cells (Fig. 1). Cells were then detached using 0.25% Trypsin-EDTA (Gibco) and centrifuged, then the pellet was collected, washed with PBS buffer, and fixed with 4% formalin (Sigma-Aldrich), then washed with distilled water. Ten microliters of the suspension from each category (control (CN), low contaminated (LC), and high contaminated cells (HC)) were deposited in spots on ZnS substrates (Crystran, UK) which were cleaned before with 70% ethanol. Cell spots were left to dry under vacuum then sealed into petri dishes to be stored until the analysis under the FTIR microscope.

### MycoAlert™ test and PCR

#### MycoAlert™ PLUS mycoplasma detection assay on infected cell supernatants

The MycoAlert™ assay was used to confirm the cells were successfully infected. This is a selective biochemical test that exploits the activity of certain mycoplasmal enzymes that react with the MycoAlert™ substrate catalyzing the conversion of ADP to ATP. By measuring the level of ATP in a sample both before and after the addition of the specific and luminescent substrate, a ratio can be obtained which is indicative of the presence or absence of mycoplasma.

One hundred microliters of cleared supernatant were transferred in a single well of 96-wells plate adding 100 μl of MycoAlert™ reagent to each sample. After 5 min, the plate was placed in a luminometer (Victor Plate Reader, Perkin Elmer) to measure luminescence (reading A). Then 100 μl of MycoAlert™ substrate were added to each sample and after 10 min luminescence was measured again (reading B). The ratio of reading B to reading A is used to determine whether a cell culture is contaminated by mycoplasma. If the value is <1, the sample is negative for mycoplasma, if it is between 1 and 1.2, the sample is borderline and the cells must be kept in quarantine and re-test in 24 h. If the ratio is more than 1.2, the sample is positive for mycoplasma contamination.

As seen in Table 1, there is a slight difference between the filtered and not filtered supernatants in all dilutions where the values of the filtered ones are lower. This is compatible with a faint retention by the pores of the 0.22-μM filter due to protein and chemical or electrostatic bond on the mesh surface that interact with the cells. The aim was to demonstrate to have obtained a mycoplasma-infected supernatant but bacteria-free. The results interpretation is based on the limit given by the MycoAlert™ detection kit as indicated by the manufacturer. For these results, no standard deviation was obtained since the manufacturer’s instructions do not suggest to perform more than one test to confirm positivity as accuracy is guaranteed at first test.

#### PCR on DBTRG cell lysate

Polymerase chain reaction (PCR) on DBTRG non-infected and infected cell lysates was performed using primers which can detect at least 49 different mycoplasma strains that include uncultured *Mycoplasma* sp. (oral), *Mycoplasma faucium*, (both from human), and many strains of *Mycoplasma bovis*. Total extract from 0.1 × 10^6^ cells/point was prepared lysing in 100 μl of 9% Nonidet-P40, 60 μg/ml proteinase K and treating at 60 °C for 30 min and 95 °C for 10 min for enzyme inactivation. PCR was performed in a SimpliAmp™ Thermal Cycler PCR System (Applied Biosystems by Thermo Fisher Scientific) for 35 cycles (30 s of denaturation at 94 °C, 30 s of annealing at 60 °C, and 60 s of elongation at 72 °C) in a volume of 25 μl reaction buffer of KAPA Taq DNA Polymerase (5 U/μl) (Kapa Biosystems by Sigma-Aldrich), 0.2 μM each primer (primer forward: 5′-ACT CCT ACG GGA GGC AGC AGT A-3′; primer reverse: 5′-TGC ACC ATC TGT CAC TCT GTT AAC CTC-3′), and 1 μl of total extract.

The possibility of contamination of infected medium by human mycoplasma strains was excluded by the negative result of Anyplex™ II STI-7 Seegene test (Seegene, Germany) that enables detection and identification of seven sexually transmitted infections pathogens, including *Mycoplasma hominis* and *Mycoplasma genitalium*, in a single real-time PCR reaction.

### FTIR data acquisition and analysis

DBTRG cell spots of each category deposited on ZnS optical substrates were analyzed in the mid-IR range on the equipment available at the MIRIAM Beamline B22 at Diamond Light Source, UK) [27]. This consists of a Vertex 80 V FTIR spectrometer (Bruker) coupled to a Hyperion 3000 microscope. The spectra were measured using the globar source with the LN2 cooled FPA detector 64 × 64 pixels (each pixel size is 40 × 40 μm^2^). Single FPA shots (field of view 128 × 128 μm^2^) were taken on many cells at a time using the 20× objective with matching condenser in transmission mode at 4 cm^−1^ spectral resolution and 256 scans accumulation (for sample and background) for a measurement time of circa 6 min. A large area of the sample was mapped with the FPA to cover many cells in order to get more chance having cells infected with mycoplasma in the contaminated series. All data acquisition was performed using the OPUS 7.2 software (Bruker). Data analysis was performed in the Unscrambler X10.3 software. Statistical analysis was performed on the PCA results using ANOVA test.

## Results and discussion

### Cell observations

Due to their small size, contamination of cell culture by mycoplasmas cannot be visualized and also it does not induce medium turbidity or color change usually generated by bacterial or fungal contamination. Moreover, morphological changes and growth rate effect induced by mycoplasmas can be minimal or even absent. In this study, the morphological changes were absent but the growth rate was slightly altered that the cells grew at a slower rate especially within the high contaminated category. Using visible microscopy the contamination of mycoplasma in the infected cells cannot be detected. As seen in Fig. 1, there is no clear difference in cell morphology or obvious bacterial presence between the infected culture (Myc+ and Myc+++) and the control non-infected DBTRG cells.

### MycoAlert™ and PCR results

**Fig. 2:**
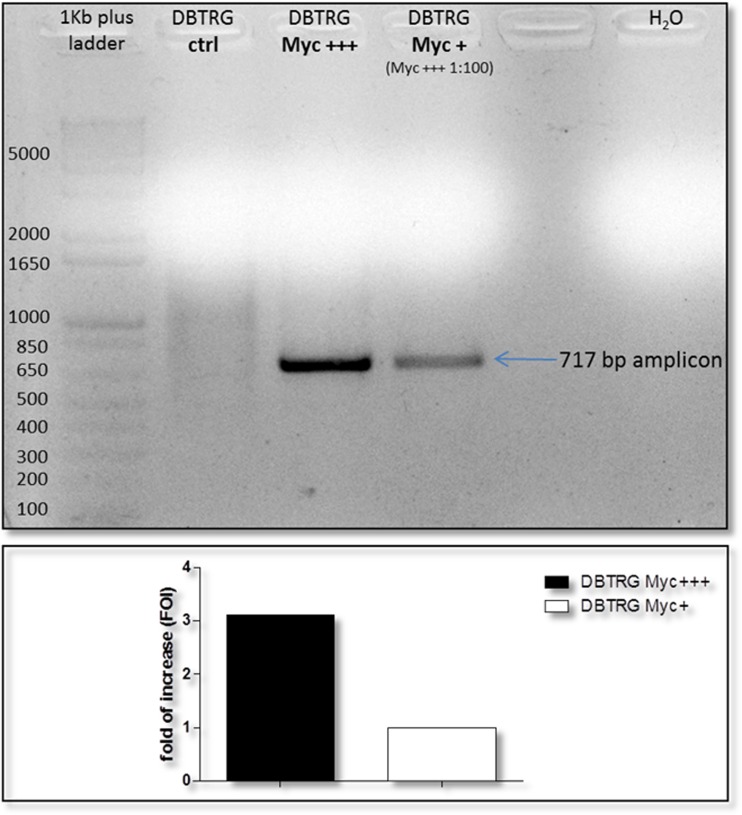
PCR detection of mycoplasma contamination on DBTRG cell lysates. Densitometry of amplicons signals reveals a threefold more mycoplasma infection in cells treated with contaminated supernatant not diluted

We demonstrated that we have successfully infected DBTRG cell line culturing them in a medium derived from a mycoplasma positive supernatant for 24 h. Both conditions, high contamination (Myc+++) and low contamination (Myc+, derived from a dilution 1:100 of Myc+++ medium), lead to an amplification of a 717-bp amplicon from 16S ribosomal RNA sequence in common with 49 different mycoplasma strains. The contamination in the lower condition approximately quantifies three times less than DBTRG cells growth in the presence of undiluted medium (Fig. 2).

The MycoAlert™ test on supernatant of DBTRG-infected cells shows that only the Myc+++ condition results are positive for mycoplasma as well as the related cell culture in PCR assay. The biochemical test seems not to reveal any positive signal in the supernatant of low contaminated cells in contrast to PCR probably less sensitive in this case (Table 2).

**Table 2:** MycoAlert™ ratio compared with PCR results in DBTRG supernatant culture and cell lysates

Supernatant from DBTRG cell culture	MycoAlert™ ratio	Result interpretation for mycoplasma contamination	Densitometry on PCR Myc amplicon on DBTRG-infected cells lysates
Myc+++ (filtered)	12.68	Positive	+++
Myc+ (filtered)	0.97	Negative	+
Negative ctrl	0.38	Negative	−

### FTIR data analysis

**Fig. 3:**
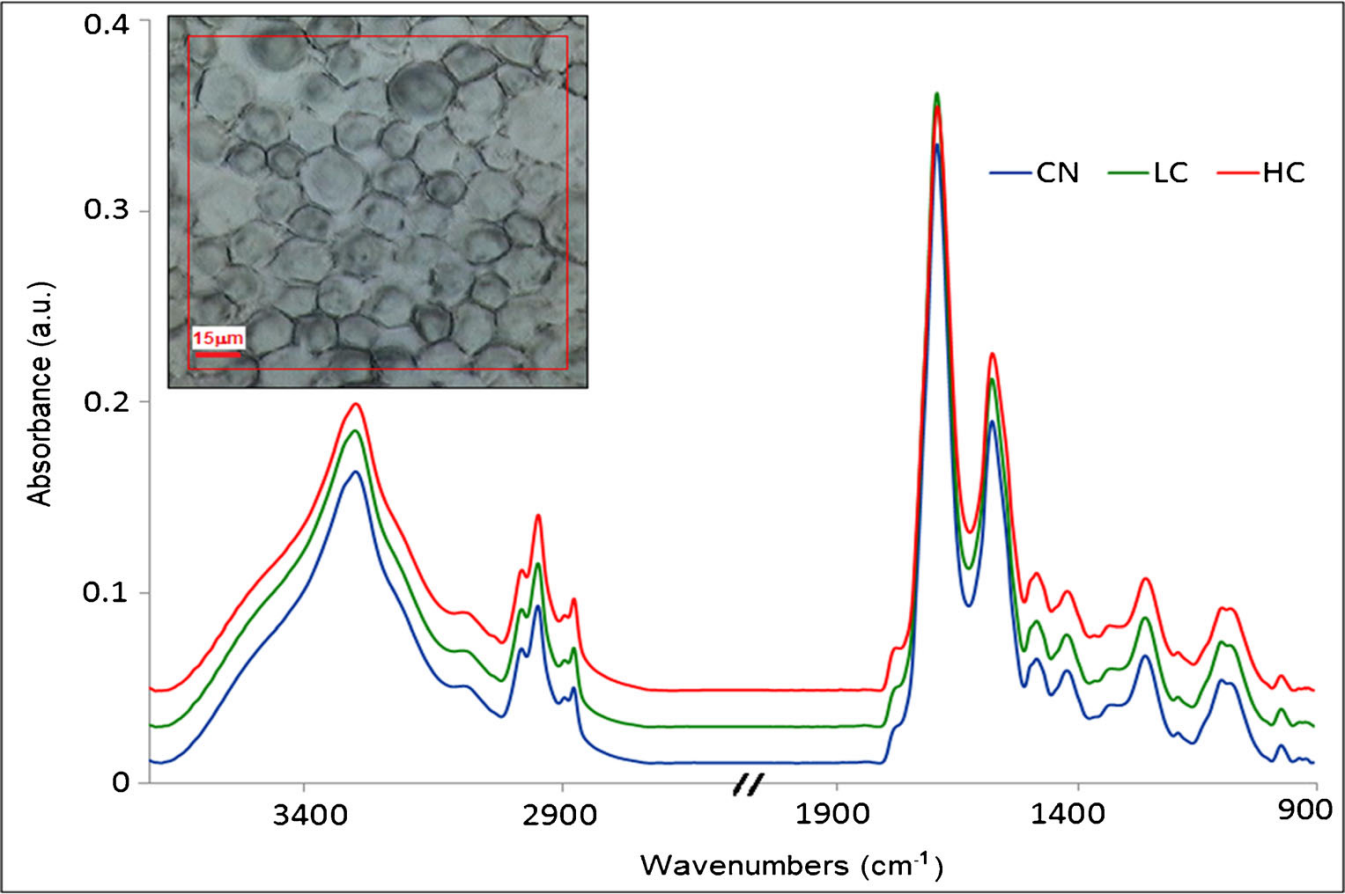
Average spectra obtained by FPA with globar source from cell spots for each category of CN (control cells, *n* = 75), LC (low contamination Myc+, *n* = 63) and HC (high contamination Myc+++, *n* = 96). Spectra (after baseline correction using concave rubberband with five iterations) were offset for clarity. Visible image of the control sample shown to the left where the red square corresponds to the FPA detector area

Spectra of the cells were retrieved from the FPA images (between 2 to 4 images per sample) and one average spectrum per cell was taken, averaging 36 to 64 pixels depending on cell size for all the cells in each category. Cells as shown in Fig. 3 are round cells obtained after trypsinization and removal from the culture flasks for preparation in suspension and deposition on ZnS substrate. The morphology is different from the ones shown in Fig. 1 which were imaged during their growth in T25 culture flasks. Some data rejection for low absorbance or noisy spectra was performed. Spectra were corrected for atmospheric compensation to remove water vapor and CO_2_ contribution. After data rejection and spectral quality control, the number of cell spectra used in the data analysis was 234 spectra (distributed for CN *n* = 75 spectra, LC *n* = 63 spectra, and HC *n* = 96 spectra). Data analysis was performed in the Unscrambler X10.3 software, using the second derivative spectra (Savitzky-Golay second order and applying 9 points smoothing) to remove slowly varying baseline effects and applying unit vector normalization. Principal component analysis (PCA) was performed on all categories grouped together, using the non-linear iterative partial least squares (NIPALS) algorithm and leverage correction validation method. Statistical analysis was performed on the PCA results using one-way ANOVA test. Average spectra were taken for each category to have an overview of the major differences in the spectra that allows the separation of the cells. Spectra were divided in separate regions to better illustrate the results and understand which are the most important bands allowing for separation among the categories. The two major regions (i.e., lipid and fingerprint) were taken into consideration, where the fingerprint region (1800–950 cm^−1^) was divided into three subregions (1800–1500, 1500–1300, and 1300–950 cm^−1^) to cover all of the membrane phospholipids, the proteins (amides I and II), the nucleic acid, and the carbohydrate absorptions.

**Fig. 4:**
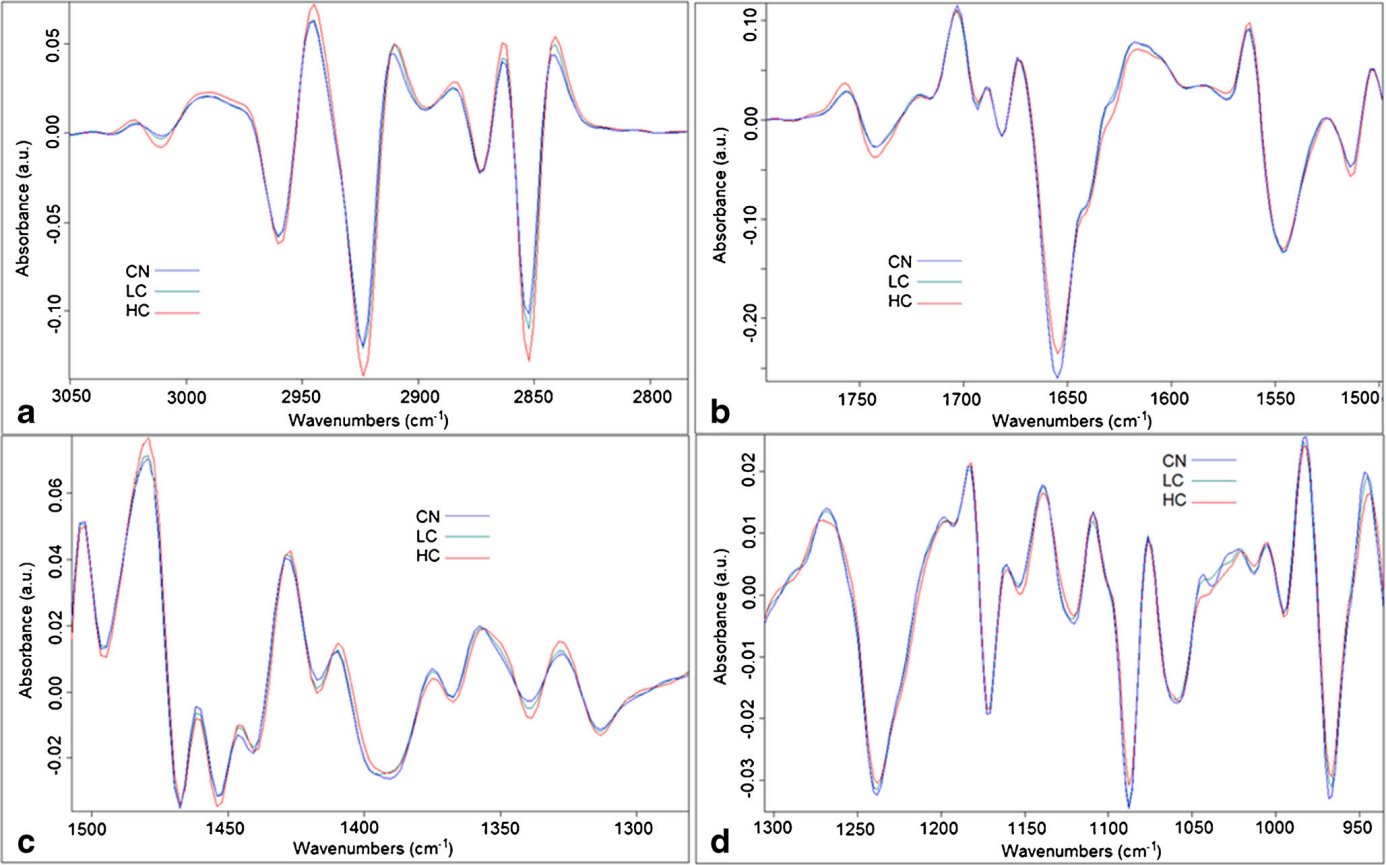
Graphs of the second derivative (9 pts smoothed and vector normalized) of the average cell spectra for each group of CN, LC, and HC. **a** Lipid region (3050–2800 cm^−1^), **b** 1760–1500 cm^−1^ region, **c** 1500–1300 cm^−1^ region, and **d** 1300–950 cm^−1^ region

The second derivative of the average spectra of each group are shown in Fig. 4 and looking at the graphs one cannot judge the major differences among the groups. There is a necessity of using a range of multivariate analyses; hence, PCA, HCA, and PCA-LDA were applied to the data to retrieve the effect of mycoplasma infection on the cells.

#### HCA analysis

**Fig. 5:**
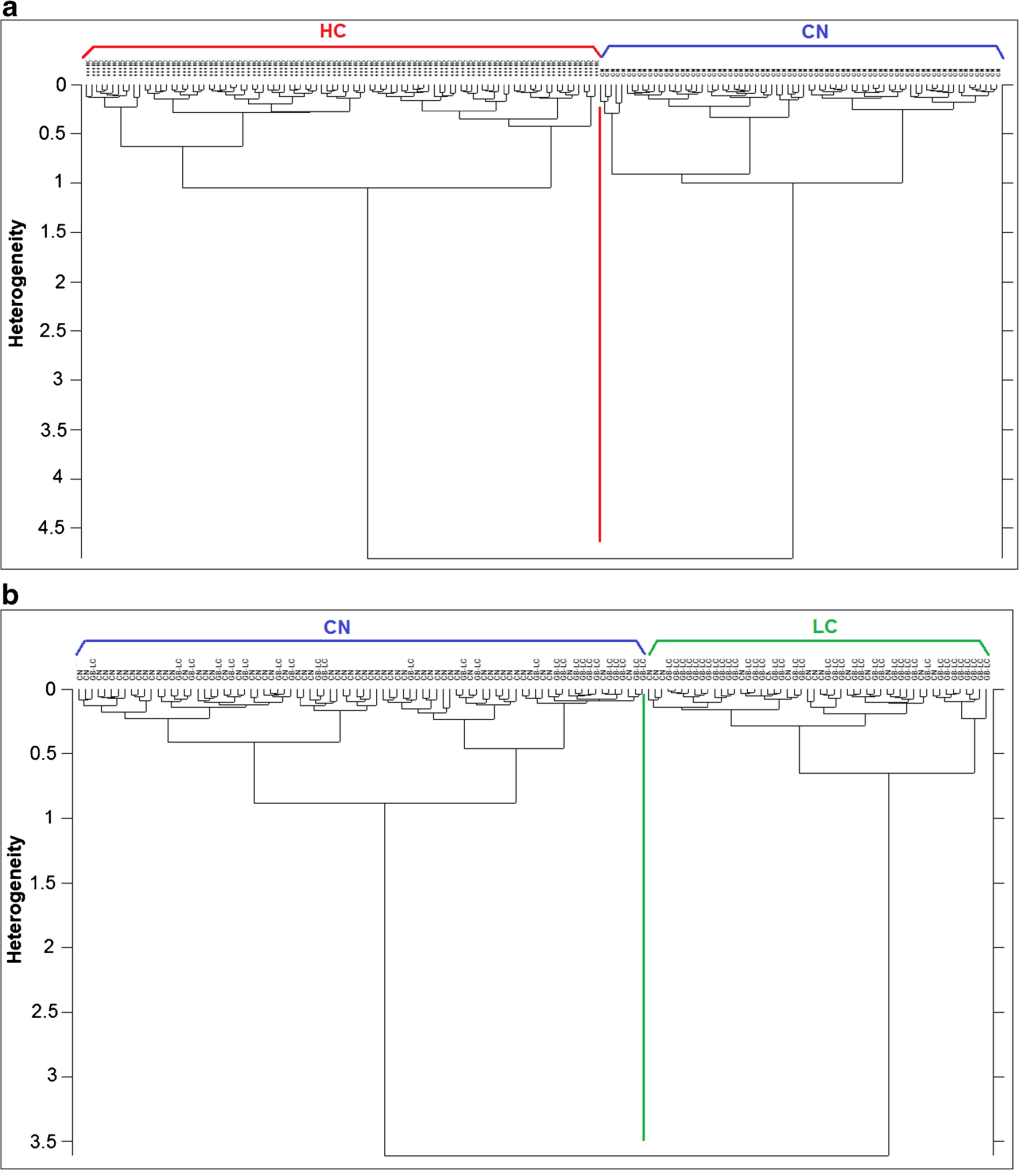

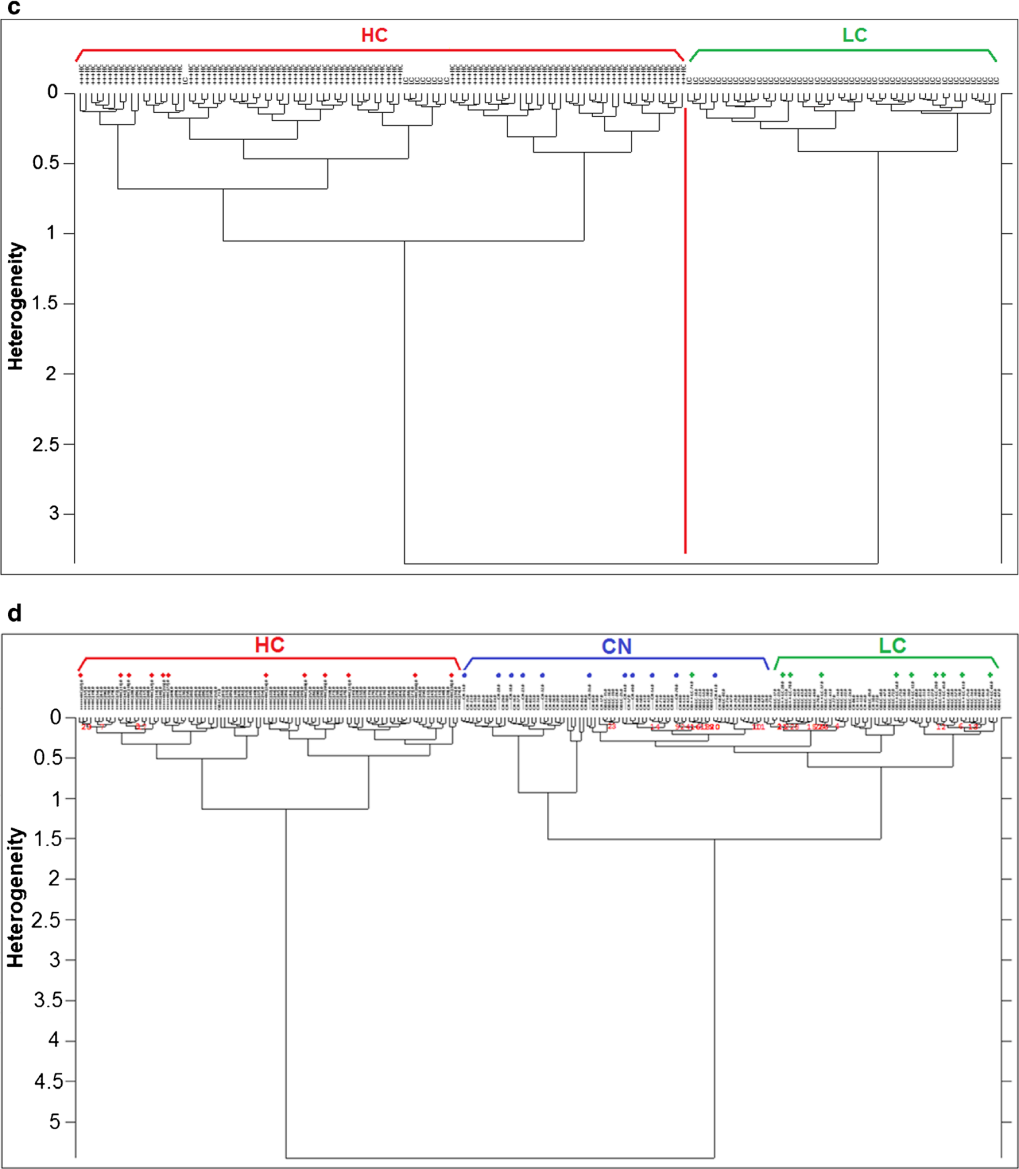
**a** HCA dendrogram of the HC vs CN groups in the 1500–1300 cm^−1^. **b** HCA dendrogram of the CN vs LC groups in the 3050–2800 cm^−1^. **c** HCA dendrogram of the HC vs LC groups in the 1500–1300 cm^−1^. **d** HCA based on the region of 1500–1300 cm^−1^ performed on second derivative and vector normalized spectra of training and test sets. Test set spectra are color-coded for better illustration and could be seen classified in the correct cluster

Hierarchical cluster analysis (HCA), an unsupervised classification method, was performed on the second derivative and vector normalized spectra of all the groups using the Ward’s algorithm to classify them into clusters. Results are shown in Fig. 5 which is divided into four parts:

- Fig. 5a*—*HCA was used to classify HC and CN groups, tried on the four spectral regions used for PCA, and the region of 1500–1300 cm^−1^ gave the best classification separating 100% the spectra.
- Fig. 5b—HCA performed on the groups CN and LC in the region of lipids 3050–2800 cm^−1^ gave better separation than the other regions. There was some overlapping which is certainly due to the low contamination in the LC group so some cells may have not been infected with the mycoplasma or affected as other cells.
- Fig. 5c—HCA dendrogram in the third part of Fig. 5 shows the separation between HC and LC groups with few cells overlapping from the LC group into the HC group. This could also be based on the degree of infection in the cells and the effects the mycoplasma entails on them. The best separation was also given by the 1500–1300 cm^−1^ spectral region.
- Fig. 5d—Since the region of 1500–1300 cm^−1^ gave the best separation in HCA between HC vs CN and HC vs LC, a test set of 33 spectra in total (11, 12, and 10 spectra from CN, HC, and LC groups, respectively) were chosen randomly to validate if this region is able to classify blindly the test set in their correct cluster within the training set (201 spectra). The results (Fig. 5d) showed very clear separation of the three groups: HC and CN spectra of the test set were classified with 100% efficiency in their correct cluster. The LC test spectra were also correctly classified in their cluster apart from one which was classified in the CN group. The test spectra are illustrated with color-coded shapes based on the class color.

#### PCA analysis

Principal component analysis (PCA), a multivariate data analysis method used to reduce dimensionality of the spectral data, was applied on the second derivative and unit vector normalized cell spectra using the non-linear iterative partial least squares (NIPALS) algorithm and leverage correction validation method. The PC loading plots are useful to understand the correlations between the variables and interpret the samples grouping as they contain the spectral features of the constituents. Peaks in the loading plots indicate important spectral regions for the particular component which are responsible for the classification. PCA shows different efficiency of separation based on the spectral region used among the four spectral regions.

##### Lipid (3050–2800 cm^−1^) spectral region

**Fig. 6:**
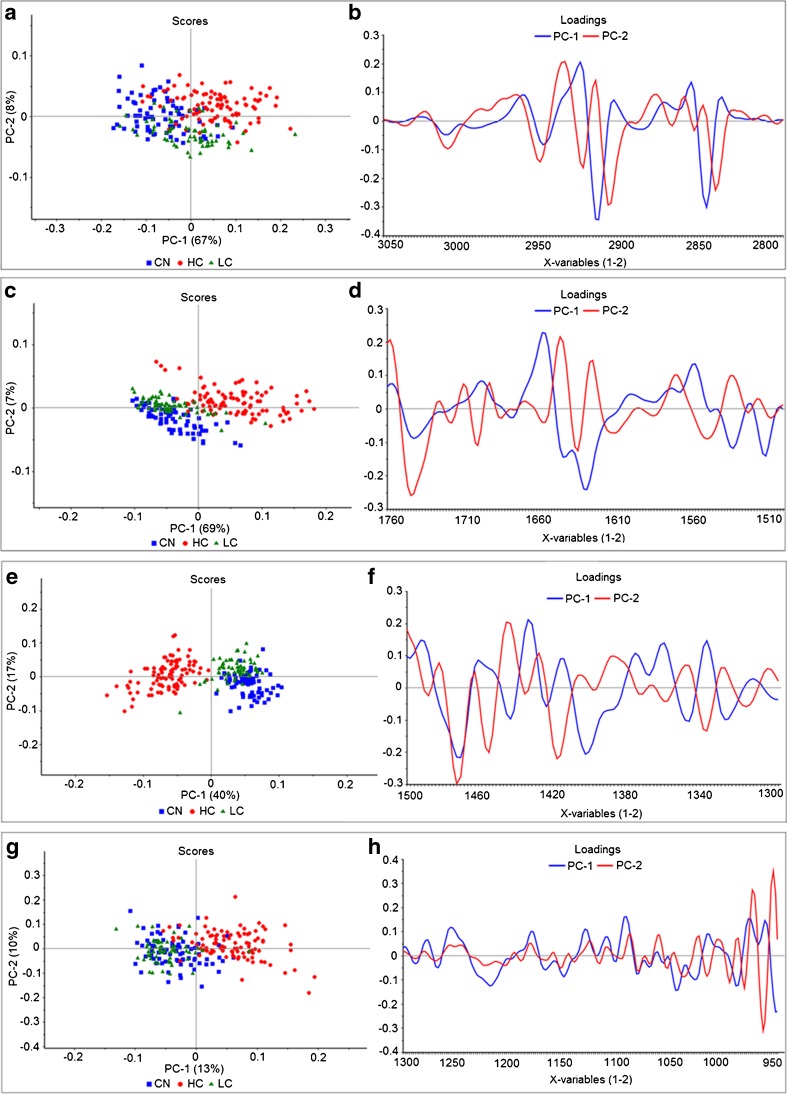
**a**–**d** PCA results for the lipid spectral region (3050–2800 cm^−1^) shown in **a** for scores and **b** for loadings and for the spectral region (1760–1500 cm^−1^) shown in **c** for scores and **d** for loadings. **e**–**h** PCA results for the spectral regions of (1500–1300 cm^−1^) shown in **e** for scores and **f** for loadings and for the spectral region (1300–950 cm^−1^) shown in **g** for scores and **h** for loadings

In Fig. 6a, PCA scores based on this region gave a good separation among the three groups. The PC loadings in Fig. 6b show the major bands explaining the difference between the groups:

PC1 separating between CN and HC mostly reveals the following bands: 3012, 2951, 2918, and 2848 on the negative side of PC1 so these intensities are lower in the CN group compared to HC (CN < LC < HC). PC2 separating between HC and LC mostly reveals bands around 3010, 2952, 2925, 2910, and 2842 on the negative side of PC2 scores which implies less intensities in the negative scores group (LC < HC).

##### 1760–1500 cm^−1^spectral region

This sub-region of the fingerprint showed also a good separation (Fig. 6c) especially in CN/LC groups vs HC group. The PC loadings in Fig. 6d show the major bands explaining the difference between the groups:

PC1 separating between CN/LC vs HC mostly reveals the following bands: 1743, 1631, 1533, and 1512 on the negative side so these intensities are lower in the CN/LC group compared to HC. PC1 loadings on the positive side reveal bands at 1658 and 1558 which are lower in HC than in CN group. PC2 mostly separating CN vs HC reveals the bands at 1745, 1700, and 1635 on the negative side so these intensities are lower in the CN group compared to HC. PC2 loadings on the positive side reveal the bands around 1647, 1625, and 1571 and imply that the intensities of these bands are lower in LC/HC groups compared to CN.

##### 1500–1300 cm^−1^spectral region

PCA scores based on this region gave the best separation among the three groups (Fig. 6e). The PC loadings in Fig. 6f show the major bands explaining the difference between the groups:

PC1 separating between HC and CN/LC mostly reveals the following bands: 1471, 1444, 1403, 1348, and 1328 on the negative side so these intensities are lower in the HC group compared to CN/LC. The positive side of PC1 loadings reveals the bands at 1434 and 1338 to be lower in CN/LC groups compared to HC. PC2 separating between LC and CN groups mostly reveals bands: 1473, 1456, 1419, and 1338 on the negative side, so the CN group which is on the negative side of PC2 scores should have less intensities of these bands than in the LC group (CN < LC). The positive side of PC2 loadings reveals the band at 1446 to be lower in LC group compared to CN group.

##### 1300–950 cm^−1^spectral region

This sub-region of the fingerprint corresponding to carbohydrates and phosphate absorption showed a good separation (Fig. 6g) between CN/LC vs HC group. The PC loadings in Fig. 6h shows the major bands explaining the difference between the groups:

PC1 and PC2 mostly reveal the following bands: 1218, 1043, 987, 981, and 964 on the negative sides of both PC loadings so these intensities are lower in the CN/LC group compared to HC. The positive side of PC1 loading reveals the bands at 1255 and 1091 to be lower in HC group compared to CN/LC group.

Since PCA results of the region 1500–1300 gave the best results in separating the three groups, more data analysis was performed on this region to evaluate the significance level statistically and understand better what is causing the differences. PCA score results in this spectral region were proven significantly different among all groups by statistical tests one way (ANOVA, *P* < 0.05). The *t* test performed on PC1 and PC2 scores for every two groups gave values < 0.01.

#### PCA-LDA analysis

Linear discriminant analysis (LDA), a supervised multivariate analysis method, is used to classify samples into groups based on features that can be used to describe the objects. Three different methods (linear, quadratic, and Mahalanobis) for the LDA can be used and the method chosen will depend on the similarity of the different classes to be discriminated. Here PCA-LDA using the Mahalanobis method was used as it may model the classes better. Since the data set contains more variables than samples (i.e., spectral data), PCA-LDA was chosen (i.e., LDA was run using the PCA scores) to overcome the dimensionality problems. The model was performed on a training set (201 spectra) using the PC scores of PC1 and PC2 of the PCA based on the spectral region 1500–1300 cm^−1^. A confusion matrix of class assignments (prediction) vs. actual classes was obtained with 87.06% accuracy; it shows the percentage of the spectra correctly classified (diagonal numbers) and misclassified (the other numbers) as illustrated in Table 3.

**Table 3:** Confusion matrix of PCA-LDA performed on training set of 201 cell spectra

Actual	CN	HC	LC
Predicted			
CN	48	0	7
HC	0	83	2
LC	16	1	44

Once the LDA model is built, it can be used to classify unknown samples or predict samples (by reflecting probability of belonging to a certain class). For this, the test samples (i.e., the same test set of 33 cell spectra used for the HCA analysis) was used and the results obtained are the following: 11 out of 11 spectra of the CN group were classified with the CN class, 11 out of 12 spectra of the HC group were classified with HC class and 1 classified with LC class, and 8 out of 10 spectra of LC group were classified with LC class and 2 with CN class.

It is noteworthy to say that the data in Table 3 shows that none of the spectra of the HC group is classed in the CN group and vice versa so no false positive or negative results were seen. This proves the accuracy of using FTIR microspectroscopy for identifying cells infected with mycoplasma. The misclassification of spectra from CN and LC groups in LC and CN classes respectively is understandable as the contamination with the mycoplasma in the LC group was very low compared to HC group so the cells of the LC group could be not all infected or that the mycoplasma did not induce yet all the effects on the cells during the short time of culture.

### Identification of spectral markers

Spectral data collected from control cells and cells which have been infected with mycoplasma led to some identification of spectral markers that could indicate the presence of mycoplasma and the effects on the host cells induced by the bacterial infection. The bands affected along with their assignments are summarized in Table 4.

**Table 4:** Bands of the differential spectrum HC-CN and their assignments [28]

FTIR bands	Assignment
3012	C-H of lipids
2951–2955	Stretching C-H (asymmetric stretching vibration of CH_3_ of acyl chains (lipids)
2918–2923	Stretching C-H (asymmetric stretching vibration of CH_2_ of acyl chains (lipids)
2848–2852	Symmetric stretching vibration of CH_2_ of acyl chains (lipids)
1743–1745	Ester group C=O of lipids (stretching vibration of phospholipids). ν(C-O) (polysaccharides, pectin)
1657	α-helical structure of amide I
1632–1635	β sheet structure of amide I. Ring C-C stretch of phenyl
1571–1557	C=N adenine. Amide II
1532	Stretching C=N, C=C
1512–1514	Stretching C=C diagnostic for the presence of a carotenoid structure, most likely a cellular pigment
1471–1477	CH_2_ bending of the methylene chains in lipids
1456	CH_3_ bending vibration (lipids and proteins)
1434 (1444–1426)	CH_2_ bending (lipids, fatty acids), CH_2_ bending (polysaccharides, cellulose)
1416 (1426–1404)	Deformation C-H, N-H, stretching C-N
1338 (1349–1328)	CH_2_ wagging. δ(CH), ring (polysaccharides, cellulose, pectin)
1313	Amide III band component of proteins
1217	PO_2_^−^ asymmetric phosphate I (nucleic acid damage?)
1090	Phosphate in nucleic acid: Phosphate I (PO_2_^−^ symmetric stretching vibration) in B-form of DNA. Phosphate II (PO_2_^−^ asymmetric stretching vibration) in A-form RNA PO_2_^−^ symmetric stretching in nucleic acid (RNA and B-form of DNA)
1044	Symmetric PO_2_^−^ stretching in RNA and DNA. C-OH group of carbohydrates (including glucose, fructose, glycogen, etc.)
961	C-O deoxyribose, C-C

To be able to identify if the spectral differences in the three groups are due to mycoplasma presence or to its effects on the host cells, an average spectrum of a positive control (dried spot taken from the mycoplasma suspension which served to infect the cells) was used to compare with the differential spectrum i.e. the subtracted spectrum of CN average from HC average (this is called HC-CN). In principle, this spectrum subtraction can highlight the bands which belong to mycoplasma presence in the highly contaminated cell samples as well as the bands in the cells which were affected by induced cellular changes due to the infection.

**Fig. 7:**
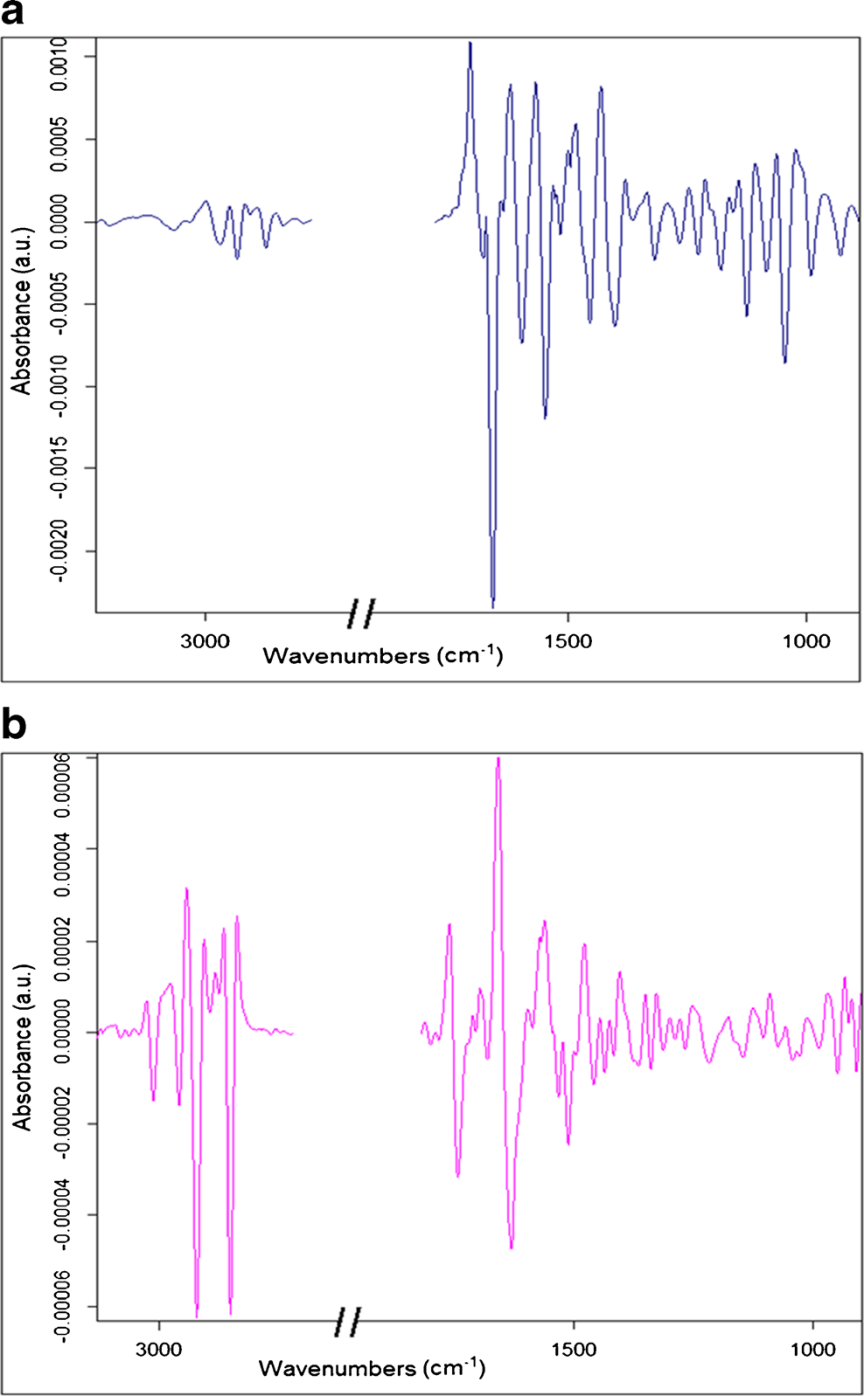
**a** Mycoplasma-positive control second derivative spectrum. **b** Differential spectrum HC-CN as a second derivative

As seen in Fig. 7, the bands for the mycoplasma suspension serving as positive control are revealed in the second derivative 9 pts smoothing of the average spectrum (Fig. 7a) and for the differential spectrum HC-CN (Fig. 7b).

In Fig. 7b, looking at the differential spectrum between HC and CN, the negative peaks (lipid region) should belong to HC and the positive peaks should belong to CN. Ignoring the common bands between mycoplasma spectrum and the differential spectrum, the responsible bands for studying the cellular changes induced by the mycoplasma on the cells can be revealed. Most of these bands are compatible with the bands revealed in PC loadings. The assignment of these bands being spectral markers is shown in Table 4 followed by the interpretation of the cellular changes due to the infection.

It was stated by Degeling et al. [1] that the effect of mycoplasma contamination depends on the particular species of mycoplasma and the contaminated cell type, i.e., some species may have no obvious effect on cellular function while many others can induce severe cytopathic changes. Since these bacteria compete with the host cells and deplete their nutrients they can cause significant effects on cellular metabolism, morphology and function [29]. Mycoplasma generation time is from 1 up to 9 h; hence, the cells were only cultured for 24 h in or w/o the presence of mycoplasma so the FTIR test sensitivity would be evaluated in the early phase of contamination and before the degeneration/loss of the cells occurs. The major known general effects of mycoplasma on eukaryotic cell cultures are the alteration of protein level, nucleic acid synthesis, alteration of cellular metabolism, chromosomal aberrations, alteration of the cellular membrane composition, interference with different biochemical and biological assays, and promotion of cellular transformation [29, 30]. The results obtained in this study confirm most if not all of these effects on the cells in the high contaminated population based on the different absorption bands revealed between the HC and CN groups. Major changes were found on membrane phospholipids, proteins, carbohydrates, and nucleic acid bands as seen in Table 4. Spectral markers were identified and could be used as mycoplasma presence indicators. The changes in lipids seen in the region of 3050–2800 cm^−1^ could be due to the change of the fatty acid components of membranes or their structural deformation. It is known that cholesterol is abundant in mycoplasmas [26], and this could also explain the higher lipid level in the HC group compared to CN. The phospholipids at 1743–1745 cm^−1^ are especially correlated with the composition of the cellular membrane being altered by the bacterial infection. The changes in the protein bands mean that the protein secondary structure is altered and deformed following the infection. Lipid and proteins content changes in the region of 1500–1300 cm^−1^ could be due to the alteration of the lipid and protein level/synthesis individually as well as the lipoprotein level as a hole. This could be easily interpreted since mycoplasmas are known to contain highly immunogenic lipoproteins anchored on the outer face of the plasma membrane [31, 32]; hence, the band at 1456 cm^−1^ for instance is lower in the control group. The changes in the phosphate bands of the nucleic acids reveal the alteration of the nucleic acid synthesis and may be chromosomal aberrations as well as structural alterations. Finally, the changes in the carbohydrates reveal mostly the alteration of the cellular metabolism.

It is noteworthy to mention that mostly the cellular membrane changes (e.g., for what concerns lipids, phospholipids, and proteins) and the protein level alteration are seen before the possible damage of nucleic acids and the alteration of the cellular metabolism. This could be concluded from the different spectral regions separating or not the LC group (not only the HC group) from the CN group. The nucleic acid region separated the HC from CN but the LC was not very well separated which also proves that the DNA region alone is not capable to identify the mycoplasma contamination. This could be one of the reasons why PCR has false negative and positive results sometimes, or why the staining with DAPI is not 100% reliable as also based on the DNA staining.

The results obtained by FTIR shows the sensitivity of the technique to detect the presence of the mycoplasma and/or the induced cellular changes by being able to differentiate the LC group from the CN group, whereas the MycoAlert™ test shows negative results for the LC group (dilution 1:100, Table 2). This is very interesting and promising that even being highly sensitive, this biochemical test could not show the positive contamination of the LC due to the detection level being slightly under the limit for the borderline. FTIR measurement was able like the PCR to detect the mycoplasma contamination in the LC group. This further proves the capabilities of FTIR to detect early biochemical changes in the cells due to the mycoplasma infection.

The preliminary results showed that FTIR has the potential to be used in the future as a reliable complementary detection technique for mycoplasma-infected cells especially that not all mycoplasma species contaminating cell cultures can be detected by a single test among the ones used nowadays due to their limited sensitivity or specificity [33]. This FTIR technique may also help to detect if cells are cleared from mycoplasmas following a treatment to eliminate the bacterial contamination, which is crucial especially for valuable cell lines that cannot be discarded when contaminated.

## Conclusion

FTIR micro-analysis was able to differentiate between the control and the mycoplasma-infected cells. Spectral markers were identified in the lipid and fingerprint regions due to molecular changes in the host cells and/or the presence of mycoplasma. Major differences are in the membrane phospholipid ester, proteins and nucleic acid regions which could be due to the cytopathic effects of mycoplasma on the host cells (e.g., disrupting the integrity of the host cell membrane and cleaving DNA and/or RNA of the host cells). Further investigations are in progress to better interpret these spectroscopic markers. The preliminary results showed that micro-FTIR has the potential to be used in the future as a complementary detection technique for mycoplasma-infected cells and could be very useful in detecting if cells are cleared from mycoplasmas following a treatment to eliminate the bacterial contamination.

## Abbreviations

FBS: Fetal bovine serum
FPA: Focal plane array
FTIR: Fourier transform infrared
MIRIAM: Multimode infrared imaging and microscopy
PBS: Phosphate-buffered saline
ZnS: Zinc sulfide
